# *Trichomonas vaginalis* induces apoptosis via ROS and ER stress response through ER–mitochondria crosstalk in SiHa cells

**DOI:** 10.1186/s13071-021-05098-2

**Published:** 2021-12-11

**Authors:** Fei Fei Gao, Juan-Hua Quan, Min A. Lee, Wei Ye, Jae-Min Yuk, Guang-Ho Cha, In-Wook Choi, Young-Ha Lee

**Affiliations:** 1 Brain Korea 21 FOUR Project for Medical Science, Chungnam National University College of Medicine, Daejeon, 35015 Korea; 2 Department of Medical Science and Department of Infection Biology, Chungnam National University College of Medicine, 6 Munhwa-dong, Jung-gu, Daejeon, 35015 Korea; 3 Department of Gastroenterology, The Affiliated Hospital of Guangdong Medical University, Zhanjiang, 524001 China; 4 Department of Obstetrics and Gynecology, Chungnam National University, DeaJeon, 35015 Korea; 5 Department of Obstetrics and Gynecology, The Affiliated Hospital of Guangdong Medical University, Zhanjiang, 524001 China

**Keywords:** *Trichomonas vaginalis*, Human cervical cancer SiHa cells, Endoplasmic reticulum stress, Mitochondrial apoptosis, Reactive oxygen species

## Abstract

**Background:**

*Trichomonas vaginalis* causes lesions on the cervicovaginal mucosa in women; however, its pathogenesis remains unclear. We have investigated the involvement of the endoplasmic reticulum (ER) in the induction of apoptosis by *T. vaginalis* and its molecular mechanisms in human cervical cancer SiHa cells.

**Methods:**

Apoptosis, reactive oxygen species (ROS) production, mitochondrial membrane potential (MMP), ER stress response and Bcl-2 family protein expression were evaluated using immunocytochemistry, flow cytometry, 5,5′,6,6′-tetrachloro-1,1′,3,3′-tetraethyl-imidacarbocyanine iodide dye staining and western blotting.

**Results:**

*Trichomonas vaginalis* induced mitochondrial ROS production, apoptosis, the ER stress response and mitochondrial dysfunction, such as MMP depolarization and an imbalance in Bcl-2 family proteins, in SiHa cells in a parasite burden- and infection time-dependent manner. Pretreatment with *N*-acetyl cysteine (ROS scavenger) or 4-phenylbutyric acid (4-PBA; ER stress inhibitor) significantly alleviated apoptosis, mitochondrial ROS production, mitochondrial dysfunction and ER stress response in a dose-dependent manner. In addition, *T. vaginalis* induced the phosphorylation of apoptosis signal regulating kinase 1 (ASK1) and c-Jun N-terminal kinases (JNK) in SiHa cells, whereas 4-PBA or SP600125 (JNK inhibitor) pretreatment significantly attenuated ASK1/JNK phosphorylation, mitochondrial dysfunction, apoptosis and ER stress response in SiHa cells, in a dose-dependent manner. Furthermore, *T. vaginalis* excretory/secretory products also induced mitochondrial ROS production, apoptosis and the ER stress response in SiHa cells, in a time-dependent manner.

**Conclusions:**

*Trichomonas vaginalis* induces apoptosis through mitochondrial ROS and ER stress responses, and also promotes ER stress-mediated mitochondrial apoptosis via the IRE1/ASK1/JNK/Bcl-2 family protein pathways in SiHa cells. These data suggest that *T. vaginalis*-induced apoptosis is affected by ROS and ER stress response via ER–mitochondria crosstalk.

**Graphical Abstract:**

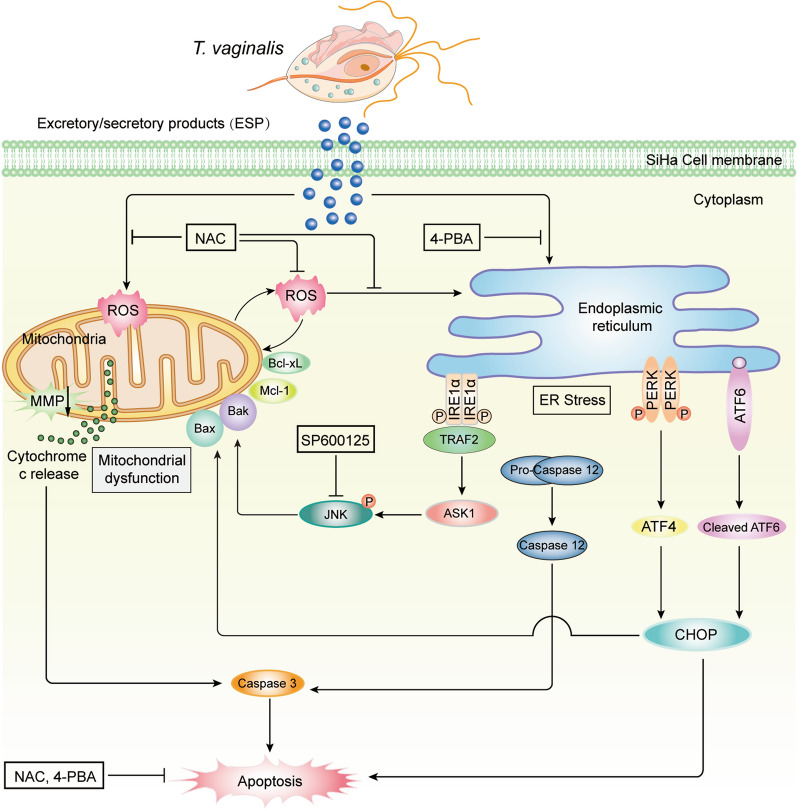

**Supplementary Information:**

The online version contains supplementary material available at 10.1186/s13071-021-05098-2.

## Background

*Trichomonas vaginalis* is a flagellated protozoan parasite that infects the female reproductive tract and the male urethra [1]. According to the 2019 report of the World Health Organization, the global prevalence estimates of trichomoniasis were 5.3% in women and 0.6% in men, and the total estimated new cases were 156.0 million (103.4–231.2 million) worldwide in 2016 [2, 3]. This parasite causes vaginitis and cervicitis in women and asymptomatic urethritis and prostatitis in men. In addition, the host inflammatory response against the parasite is predicted to result in multiple adverse health effects, such as a high incidence of premature births [4] and increased risk of cervical cancer [5]. However, the pathogenesis for *T. vaginalis* infection is poorly understood.

Apoptosis, a cellular event induced by the activation of a series of enzymes known as caspases, occurs when a cell is damaged beyond repair, infected with a pathogen or stressed due to DNA damage or toxic chemicals [6]. Mitochondria are important in the regulation and transmission of apoptotic signals, which are regulated by maintaining a balance among the levels of the Bcl-2-family proteins [7]. Several studies have presented various mechanisms underlying the induction of apoptosis by *T. vaginalis* infection using in vitro models [8–14]. *Trichomonas vaginalis*-induced apoptosis of cells has been described via the production of reactive oxygen species (ROS) [8, 9], secretion of cysteine proteases from *T. vaginalis* [10–12] and mitochondrial dysfunction, including an altered mitochondrial membrane potential (MMP) and an imbalance in Bcl-2 family protein expression [13, 14]. However, the role of the endoplasmic reticulum (ER) in *T. vaginalis*-induced apoptosis has not been elucidated.

ROS function as intermediates in cellular processes, such as inflammatory responses, cell-cycle progression, apoptosis, aging and cancer, and are produced in various organelles and via different enzyme systems, including mitochondria, ER, peroxisomes and NADPH oxidase [15]. The mitochondrion is a major source of ROS [16]. Previous reports have shown that ROS induce apoptosis in *T. vaginalis-*infected human neutrophils and cervical cancer cells through caspase-3 activation and nuclear factor kappa B (NF-κB) inactivation, respectively [8, 9]. ROS also activate NLRP3 inflammasome formation in human prostate epithelial RWPE-1 cells [17]. However, the interaction between ROS and ER stress in *T. vaginalis-*infected cells has not been studied.

The ER plays a role in protein folding and assembly, lipid biosynthesis, vesicular traffic and cellular calcium storage [18]. Its function can be disturbed by various factors, including the expression of misfolded or unfolded proteins, changes in calcium homeostasis, redox status and energy storage. These dysfunctions cause proteotoxicity in the ER, collectively termed ER stress, which leads to activation of the unfolded protein response (UPR) [18]. ER stress induces dissociation of the chaperone glucose-regulated protein 78 (GRP78) from the three ER transmembrane protein stress sensors, named inositol-requiring enzyme (IRE), protein kinase RNA (PKR)-like ER kinase (PERK) and activating transcription factor 6 (ATF6). IRE1α is an ER transmembrane protein that activates the UPR to maintain ER and cellular function. PERK is a trans-ER membrane serine/threonine kinase which is activated by misfolded proteins in the ER lumen. Phospho (p)-PERK activates eukaryotic initiation factor-2α (eIF2α), which suppresses protein synthesis. ATF6 is a transmembrane glycoprotein, and is cleaved after the accumulation of misfolded proteins in the ER. Activation of these three protein sensors induces the UPR, which increases the expression of CCAAT/enhancer-binding protein–homologous protein (CHOP). CHOP is a proapoptotic transcription factor that plays an important role in ER stress-induced apoptosis. Caspase-12 is a prodeath protease located on the outer surface of the ER membrane that is responsible for ER stress-induced apoptosis [18, 19].

Uncontrolled severe oxidative stress triggers a series of proapoptotic signaling pathways, including the ER stress response and mitochondrial dysfunction, ultimately resulting in cell apoptosis [20]. Many researchers have reported the induction of ER stress responses in cells infected by bacteria [21, 22]. ER stress has also been investigated in cells infected by protozoan parasites, including *Plasmodium*, *Toxoplasma*, *Cryptosporidium* and *Leishmania* [23]. However, ER stress induction in cells infected with *T. vaginalis* has not yet been explored. Therefore, in the present study, our aim was to study the involvement of ER stress response in apoptosis induction, as well as its potential molecular mechanisms, in *T. vaginalis*-infected human cervical cancer SiHa cells. Thus, we evaluated the cytotoxicity, apoptosis, ROS production, MMP, ER stress response and Bcl-2 family protein levels in *T. vaginalis*-infected SiHa cells with or without specific inhibitors using lactate dehydrogenase (LDH) assays, immunocytochemistry, flow cytometry, 5,5′,6,6′-tetrachloro-1,1′,3,3′-tetraethyl-imidacarbocyanine iodide (JC-1) staining and western blotting.

## Methods

### Reagents and antibodies

The CytoTox 96® Non-Radioactive Cytotoxicity Assay was obtained from Promega (Madison, WI, USA). Life Technologies CellROX® oxidative stress reagents (Thermo Fisher Scientific, Waltham, MA, USA) were purchased from Alfagene Ltd. (Carcavelos, Portugal). The MitoSOX™ Red Mitochondrial Superoxide indicator was purchased from Thermo Fisher Scientific. The JC-1 MitoMP detection kit was obtained from Dojindo Laboratories (Kumamoto, Japan). The FITC Annexin V Apoptosis Detection Kit was purchased from BD Biosciences (San Diego, CA, USA). The ER stress inhibitor 4-phenylbutyric acid (4-PBA), the ROS scavenger* N*-acetyl-l-cysteine (NAC) and the C-Jun N-terminal kinase (JNK) inhibitor SP600125 were purchased from Sigma Chemical Co. (St. Louis, MO, USA).

ER Stress Antibody Sampler Kit, anti-ATF6, anti-p-PERK, anti-phospho eukaryotic translation initiation factor 2α (ani-p-eIF2α), anti-poly (ADP-ribose) polymerase (anti-PARP), anti-cleaved caspase-3, anti-caspase-3, the pro-Apoptosis Bcl2 Family Member Antibody Sampler Kit, the pro-Survival Bcl2 Family Member Antibody Sampler Kit, anti-phospho-apoptosis signal-regulating kinase 1 (anti-p-ASK1, Ser967), anti-phospho-ASK1 (Thr845), anti-ASK1, anti-phospho-JNK (anti-p-JNK, Thr183/Tyr185), anti-JNK (56G8) and anti-β-actin antibodies were purchased from Cell Signaling Technology Inc. (Danvers, MA, USA). Anti-CHOP and anti-phospho-IRE1α (anti-p-IRE1α) antibodies were obtained from Abcam (Cambridge, MA, USA). The secondary antibodies anti-rabbit horseradish peroxidase (HRP) and anti-mouse HRP were obtained from Jackson Immuno Research Laboratories (West Grove, PA, USA). Goat anti-Mouse IgG (H + L) Highly Cross-Adsorbed Secondary Antibody–Alexa Fluor 568 was obtained from Thermo Fisher Scientific.

### Culture of SiHa cells

Cells of the human cervical cancer cell line SiHa (ATCC® HTB-35™) was obtained from the American Type Culture Collection (ATCC, Manassas, VA, USA) and maintained in Dulbeco’s Modified Eagle’s Medium (DMEM) supplemented with 10% heat-inactivated fetal bovine serum (FBS; Gibco BRL, Grand Island, NY, USA) and antibiotic–antimycotic (Gibco BRL) in a 5% CO_2_ atmosphere at 37 ˚C.

### *Trichomonas vaginalis* cultures

The *T. vaginalis* T016 strain was cultured according to previously reported methods [8, 12]. Briefly, *T. vaginalis* T016 isolate was cultured in screw-capped glass tubes containing Diamond’s trypticase yeast-extract maltose (TYM) medium (NAPCO, Winchester, VA, USA) supplemented with 10% heat-inactivated horse serum (Sigma-Aldrich, St Louis, MO, USA) in 5% CO_2_ at 37 ˚C for 24 h. The cultured parasites were monitored for motility, and their viability was determined before each experiment by staining with trypan blue (> 99%).

### Preparation of* T. vaginalis* excretory/secretory products

*Trichomonas vaginalis* excretory/secretory products (ESP) was prepared as described previously [12]. To prepare the *T. vaginalis* ESP, freshly purified trophozoites (1 × 10^7^ cells/mL) were incubated with TYM medium at 37 °C for 1 h in 5% CO_2_. After centrifugation for 30 min at 10,000 *g*, the ESP-containing supernatant was filtered through a 0.2-μm pore. The *T. vaginalis* ESP concentrations were measured by the Bio-Rad DC Protein Assay (Bio-Rad Laboratories, Inc., Hercules, CA, USA) with bovine serum albumin (BSA) as the standard. The samples were kept at − 70 °C until use.

### Experimental designs

SiHa cells were seeded on 96-well plates [for the LDH and 3-(4,5-dimethylthiazol-2-yl)-5-(3-carboxymethoxyphenyl)-2-(4-sulfophenyl)-2H-tetrazolium, inner salt [MTS] assays), on 12-well coverslips for immunofluorescence and ROS detection and in 100-mm culture dishes (for western blotting), at various densities and grown to confluence at 37 °C in 5% CO_2_.

Based on our previous studies [9, 12], live *T. vaginalis* trophozoites were incubated in SiHa cells in mixed-medium (DMEM:TYM, 2:1) at a multiplicity of infection (MOI) of 2 or 5 for 2 and 6 h, at 37 °C in 5% CO_2_. Following the predetermined incubation time, cytotoxicity, apoptosis, ROS production, MMP, induction of ER stress and its mechanism of action and Bcl-2 family-related protein expressions were evaluated in *T. vaginalis*-infected SiHa cells by the MTS assay, LDH assay, flow cytometry, immunocytochemistry and western blotting.

To determine the role of ROS in *T. vaginalis*-infected cells, cells were pretreated with the ROS scavenger NAC and then evaluated for apoptotic features, MMP and ER stress response. To assess the involvement of ER stress in apoptosis induction in *T. vaginalis*-infected SiHa cells, cells were pretreated with ER stress inhibitor 4-PBA, NAC or the JNK inhibitor SP600125, and then the apoptotic features, ROS production, MMP, Bcl-2 family proteins and ASK1/JNK pathways were evaluated. We also checked the induction of mitochondrial ROS, apoptosis and ER stress response in SiHa cells after treatment with *T. vaginalis* ESPs. Untreated SiHa cells were used as controls. Each experiment was performed at least three times in triplicate.

### LDH assay

The LDH assay was performed for cytotoxicity quantification with the CytoTox 96® Non-Radioactive Cytotoxicity Assay kit (Promega) according to the manufacturer’s protocol. Briefly, 1 × 10^4^ cells were seeded in 96-well plates and infected with *T. vaginalis* at various MOI (1, 2, 5, 10) for the indicated times (0, 0.5, 2, 6, 12 and 24 h) in an incubator (5% CO_2_, 90% relative humidity, 37 °C). Then, 50 µl of the culture supernatant from all infected and control wells was transferred into a new 96-well plate, and 50 µl of CytoTox 96® reagent was added to each sample aliquot. The plate was covered with foil to protect the samples from light and incubated for 30 min at room temperature. After adding 50 µl of stop solution to each well, the absorbance of the solution was measured immediately at 490 nm using a microplate reader (TECAN, Crailsheim, Germany). LDH release levels in the media were quantified and compared to maximum LDH release values according to the kit instructions.

### MTS assay

The MTS assay was performed to detect cytotoxicity with the CellTiter 96® AQueous One Solution Cell Proliferation Assay kit according to the manufacturer’s instructions. Briefly, 1 × 10^4^ SiHa cells were seeded in 96-well plates and stimulated with various concentrations of NAC (0, 0.2, 1, 5 mM), 4-PBA (0, 0.2, 1, 2 mM) and SP600125 (0, 0.3, 3, 30 µM) for the indicated times (0, 2, 6, 12, 24 h) in an incubator (5% CO_2_, 37 °C). Then, 20 µl of CellTiter 96® AQueous One Solution Reagent was pipetted into each well of the 96-well assay plate containing the samples in 100 µl of culture medium, following which the samples were incubated at 37 °C for 1–4 h in a humidified, 5% CO_2_ atmosphere and the absorbance recorded at 490 nm using a 96-well plate reader.

### Measurement of ROS generation by confocal microscopy

SiHa cells (1 × 10^4^ cells/well; 12-well plates) seeded on a coverslip were cultured in DMEM supplemented with 10% FBS and the culture medium replaced when the cells reached 80% confluence. To evaluate the generation of ROS, SiHa cells were first infected with live *T. vaginalis* (MOI 2 and 5) or treated with 100 µg/ml *T. vaginalis* ESP for 2 and 6 h, then incubated with 5 µM MitoSOX reagent or CellROX reagent and finally incubated for 10 min at 37 °C, 5% CO_2_ in the dark. The stained cells were imaged using a laser confocal microscope (model TCS SP8; Leica Microsystems GmbH, Wetzlar, Germany). All experiments were performed on triplicate samples, and fluorescence intensity was calculated using ImageJ software; the graph was plotted using SigmaPlot version 12.5 (Systat Software Inc., San Jose, CA, USA).

### ROS-based flow cytometric assay

SiHa cells were cultured in 12-well plates and then treated with 100 µg/ml *T. vaginalis* ESP for 2 and 6 h or infected with *T. vaginalis* at MOI 2 and 5 for 0, 2 and 6 h; the cells were pretreated or not with the indicated concentrations of NAC (0, 0.2, 1, 5 mM). The cells were then washed with phosphate-buffered saline (PBS), removed from the well with 2.5% trypsin–EDTA and re-suspended in PBS with 5 µM MitoSOX reagent or 5 µM CellROX reagent and incubated at 37 ℃, protected from light, for 30 min. After gentle washing (3 times), the cells were re-suspended in FACS buffer (1% BSA in PBS) and immediately subjected to the acquisition of 10,000 events by a FACScan instrument (BD Biosciences). The results were expressed as a histogram emitting the corresponding fluorescence and as a bar graph representing the mean fluorescence intensity of all the groups.

### MMP assay

The MMP was measured using the JC-1 MitoMP Detection Kit (Dojindo, Kumamoto, Japan). Briefly, SiHa cells were seeded onto coverslips in 12-well plates at a density of 1 × 10^4^ cells/well and infected with *T. vaginalis* at various conditions with or without specific inhibitors. The cells were then incubated with 4 μM JC-1 fluorescence dye at 37 °C for 30 min in dark and rinsed three times with Hanks' Balanced Salt Solution (HBSS). The stained cells were mounted onto microscope slides in VECTASHIELD HardSet Mounting Medium containing 4′,6-diamidino-2-phenylindole (DAPI; Vector Laboratories, Inc., Burlingame, CA, USA), and images were collected using a laser confocal microscope (model TCS SP8; Leica Microsystems GmbH). The intensities of green (excitation/emission wavelength: 485/538 nm) and red (excitation/emission wavelength: 485/590 nm) fluorescence were analyzed for ≥ 6 microscopic fields in each sample.

### Immunocytochemistry

SiHa cells were seeded onto coverslips in 12-well plates at a density of 1 × 10^4^ cells/well and incubated for 24 h. The cells were pretreated with or without specific inhibitors for 2 h and then infected with *T. vaginalis* at MOI 5 for 6 h. The cells were washed with HBSS and fixed with freshly prepared 4% paraformaldehyde for 1 h at room temperature, then washed three times in PBS containing 0.3% Triton X-100 (PBS-T) for 5 min. The cells were then blocked with 1% BSA in 0.3% PBS-T for 30 min at room temperature, incubated with CHOP primary antibody for 2 h at room temperature, washed to remove the excess primary antibody and then incubated with the appropriate fluorescently labeled secondary antibody (anti-mouse Alexa Fluor 647) for 2 h at room temperature. After mounting with VECTASHIELD HardSet Antifade Mounting Medium with DAPI (Vector Laboratories, Inc.) to stain the nucleus, fluorescence images were acquired using a confocal microscope (Leica Microsystems GmbH).

### Western blotting analysis

Sodium dodecyl sulfate-polyacrylamide gel electrophoresis (SDS-PAGE) and western blotting analysis were performed to determine the expression of numerous proteins. The 1 × 10^7^ SiHa cells were cultured with complete medium (DMEM supplemented with 10% FBS and 1 × antibiotic–antimycotic) in 100-mm dishes and then subjected to serum deprivation for 4 h to remove any stimulation from serum factors. The SiHa cells were then treated with 100 µg/ml *T. vaginalis* ESP for 0, 2 and 6 h or infected by *T. vaginalis* at various MOIs (1, 2, 5 and 10) for the indicated times (0, 0.5, 2, 6, 12 and 24 h). SiHa cells were pretreated with various concentrations of NAC (0, 0.2, 1, 5 mM), 4-PBA (0, 0.2, 1, 2 mM) and SP600125 (0, 0.3, 3, 30 µM) for 2 h, and subsequently infected with *T. vaginalis* at MOI 5 for 6 h. After washing with PBS, proteins were extracted using the PRO-PREP Protein Extraction Solution (iNtRON Biotechnology, Seongnam, Gyeonggi, Korea). The extract was incubated with the complete protease inhibitor cocktail (Roche, Basel, Switzerland) for 30 min on ice followed by boiling for 10 min, and then centrifuged at 14,000 *g* for 15 min at 4 °C. The supernatant was collected, and equal amounts of protein from different samples were separated by SDS-PAGE and transferred to a polyvinylidene difluoride (PVDF) membrane (Bio-Rad Laboratories, Hercules, CA, USA). The membranes were immersed for blocking at 5% skim milk in Tris-buffered saline (20 mM Tris, 137 mM NaCl, pH 7.6) containing 0.1% Tween-20 (TBST) for 1 h at room temperature. After one washing in TBST, the membranes were incubated overnight at 4 °C with the primary antibodies diluted in TBST supplemented with 5% BSA (1:1000). Following three consecutive washes with TBST, the membranes were incubated for 90 min with HRP-conjugated anti-mouse or anti-rabbit secondary antibody diluted 1:2000 with 5% skim milk, as described above. The washed membranes were soaked with Immobilon Western Chemiluminescent HRP Substrate (Jackson ImmunoResearch Laboratories Inc., West Grove, PA, USA), and chemiluminescence was detected with a Fusion Solo System (Vilber Lourmat, Collegien, France). Band intensity was quantified using ImageJ software (NIH, Bethesda, MD, USA). These experiments were repeated at least three times.

### Apoptosis detection by FITC Annexin V/propidium iodide

We used the BD Biosciences FITC Annexin V Apoptosis Detection Kit to measure *T. vaginalis*-induced SiHa cell apoptosis. Briefly, the SiHa cells were plated into a 6-well plate and infected with *T. vaginalis* at MOI 2 and 5 for the indicated times (0, 2 and 6 h), following which both floating and adherent cells were collected and washed twice with cold PBS. The cells were then resuspended in 1 × Binding Buffer at a concentration of 1 × 10^6^ cells/ml, and 100 µl of the solution (1 × 10^5^ cells) was transferred to a new FACS tube and 5 µl of FITC Annexin V and 5 µl of propidium iodide (PI) were added. The cells in the FACS tube were then gently vortexed and incubated for 15 min at room temperature in the dark. After 400 µl of 1 × Binding Buffer was added to each FACS tube, the cells were analyzed using a FACSCanto II flow cytometer (BD Biosciences) within 1 h.

### Statistical analysis

All assays were performed in triplicate, and at least three independent experiments were conducted per test series. The results are presented as the mean ± standard deviation (SD). Statistical analysis of the data was performed using unpaired, two-tailed Student’s *t*-tests. A *P* value < 0.05 indicated statistical significance.

## Results

### *Trichomonas vaginalis*-induced cytotoxicity and mitochondrial ROS production in SiHa cells in a parasite burden- and infection time-dependent manner

To determine the adequate MOI and infection time for induction of apoptosis and ER stress response in live *T. vaginalis*-infected SiHa cells, we performed the LDH assay and western blotting under various conditions. LDH-dependent cytotoxicity was significantly increased by 30 min after infection and increased with increasing MOIs in SiHa cells between 2 and 6 h after infection (see Additional file 1: Figure S1a). The levels of apoptosis- and ER stress-related proteins also increased by 30 min after infection, and their protein bands were apparent at 2 and 6 h after infection. However, the band densities of some protein bands were reduced from 12 h after infection onwards. Among the various MOIs of live *T. vaginalis* parasites tested, the expressions of apoptosis- and ER stress-related proteins changed in a dose-dependent manner at MOI 2 and 5 (see Additional file 1: Figure S1b).

**Fig. 1:**
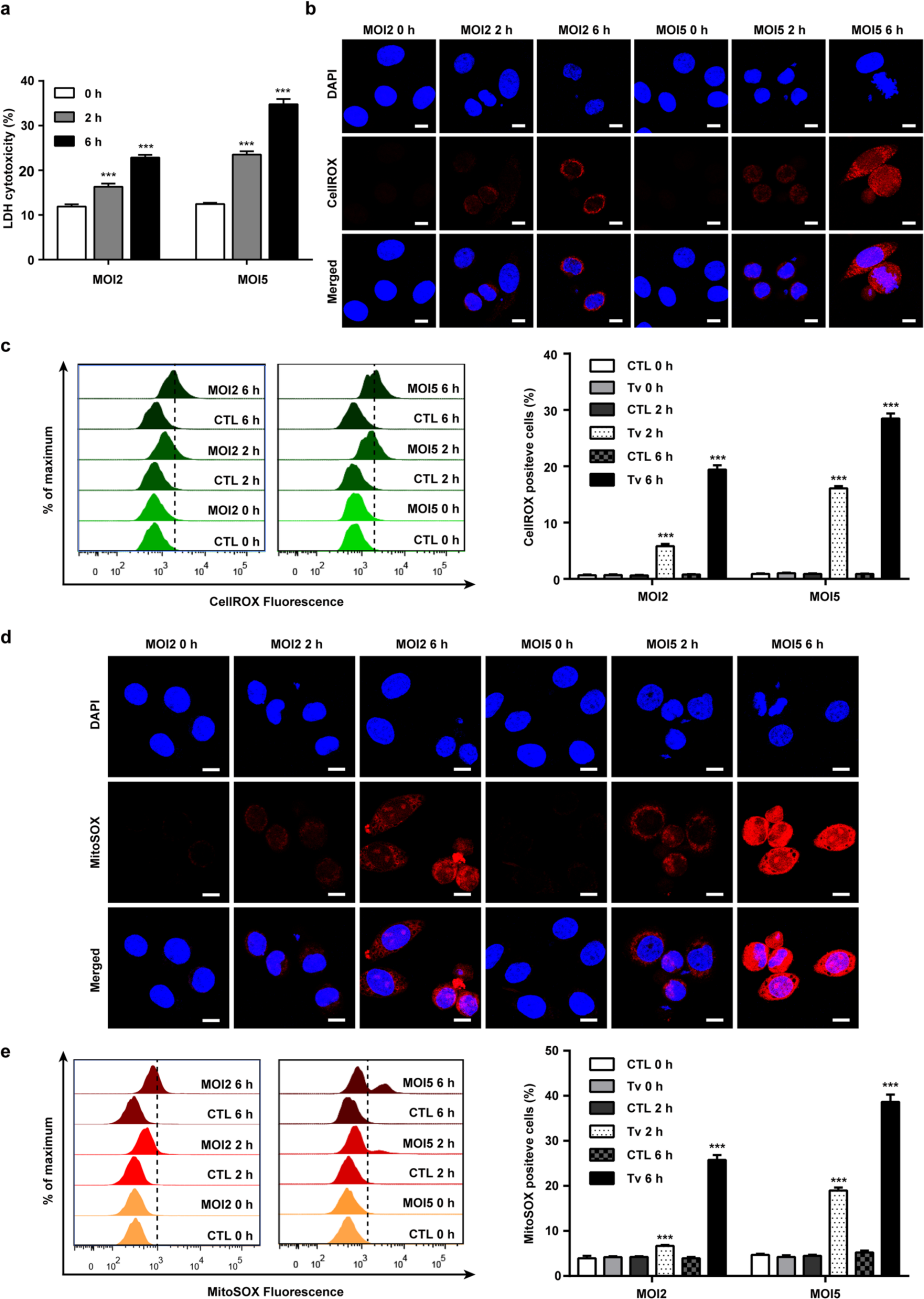
*Trichomonas vaginalis*-induced cytotoxicity and mitochondrial ROS production in SiHa cells. SiHa cells were infected with live *T. vaginalis* trophozoites at MOI 2 and 5 for 0, 2 or 6 h. **a** The LDH level in the medium, which is related to cell death, was measured by LDH assay at the indicated conditions. The data represent the mean value ± standard deviaiton (SD) of at least three independent experiments. Asterisks indicate significant difference (***P < 0.001) compared with untreated control cells under the same conditions. **b, c** Cellular ROS production was measured with CellROX reagent, a fluorogenic probe for measuring oxidative stress in live cells by confocal microscopy (**b**) and flow cytomery (**c**). **d**, **e** Mitochondrial ROS production was determined by confocal microscopy (**d**) and flow cytometry with MitoSOX, a mitochondrial ROS dye (**e**). Plots (**c**, **e**) depict the CellROX or MitoSOX-positive cells as determined by fluorescence analysis of flow cytometry results. Data shown are representative of three independent experiments with similar results. Asterisks indicate significant difference (***P < 0.001) in mean fluorescence compared with untreated control cells under the same conditions Scale bars: 10 µm. Abbreviations: CTL, Untreated control SiHa cells; Tv, SiHa cells infected with live *T. vaginalis*

Based on the results shown in Additional file 1 and from our previous studies [9, 12], we selected MOI 2 and 5 as an adequate parasite burden, and 2 and 6 h as an adequate *T. vaginalis* infection period to induce apoptosis and the ER stress response. The cytotoxicity of SiHa cells infected with *T. vaginalis* increased with the MOI and in a time-dependent manner (Fig. 1a). As oxidative stress is an important factor in the cytotoxicity associated with apoptosis [15, 16], we checked the cellular and mitochondrial ROS levels in *T. vaginalis*-infected SiHa cells. Cellular ROS levels were significantly increased in SiHa cells (MOI 2) from 2 h after infection and had increased further at 6 h post-infection. Cellular ROS levels were significantly higher in cells infected with *T. vaginalis* at MOI 5 than in cells infected with *T. vaginalis* at MOI 2 (Fig. 1b, c). Similarly, mitochondrial ROS production in the SiHa cells significantly increased from 2 h after infection, and increased in direct proportion to parasite burden and infection time (Fig. 1d, e). These findings suggested that *T*. *vaginalis* induces cytotoxicity and mitochondrial ROS production in SiHa cells in a parasite burden- and infection time-dependent manner.

### *Trichomonas vaginalis-*induced mitochondrial apoptosis through ROS in SiHa cells

**Fig. 2:**
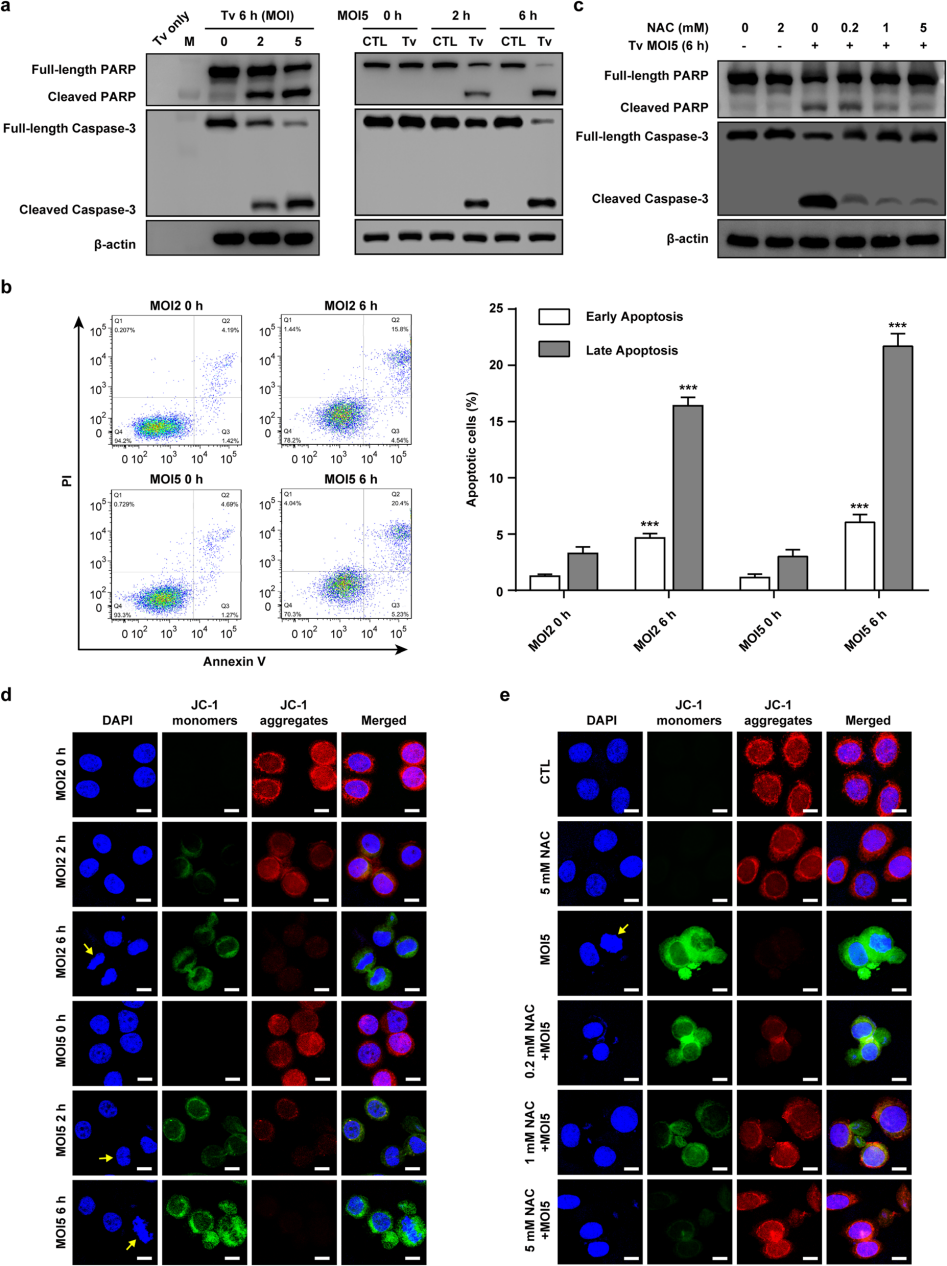
*Trichomonas vaginalis* induced mitochondrial apoptosis in SiHa cells. SiHa cells were infected with live *T. vaginalis* trophozoites at MOI 2 and 5 for 0, 2 or 6 h. **a** PARP and caspase-3 proteins were assessed using western blotting analysis, and anti-β-actin was used as a loading control. Lanes: Tv only, *T. vaginalis* parasite protein without SiHa cells; M, protein marker; CTL, untreated control SiHa cells; Tv, SiHa cells infected with live *T. vaginalis*. **b** Ratio of apoptosis was measured and analyzed by Annexin V-FITC/PI staining and flow cytometry. Cells that are considered to be viable are both FITC Annexin V and propidium iodide (PI) negative; cells that are necrotic cells are FITC Annexin V negative and PI positive; cells that are in early apoptosis are FITC Annexin V positive and PI negative; and cells that are in late apoptosis or already dead are are both FITC Annexin V and PI positive. Data are presented as the means ± SD, and asterisks indicate significant difference (***P < 0.001) compared to the control group. **c** SiHa cells were pretreated with various concentrations of an ROS scavenger (NAC) for 2 h, and subsequently infected with *T. vaginalis* at MOI 5 for 6 h. PARP and cleaved caspase 3 protein levels were assessed by western blotting. **d** JC-1 staining was observed by confocal fluorescence microscopy. In JC-1-stained cells, red fluorescence is visible in cells with high mitochondrial membrane potential, while green fluorescence of JC-1 monomer is present in cells with low mitochondrial potential. **e** SiHa cells were pretreated with various concentration of NAC for 2 h and subsequently infected with *T. vaginalis* at MOI 5 for 6 h. JC-1 staining was observed using confocal fluorescence microscopy. Scale bars: 10 µm. Data shown are representative of three independent experiments with similar results

To determine whether the observed cytotoxicity is associated with apoptosis, we conducted western blotting and flow cytometry to detect apoptotic signals in the *T. vaginalis*-infected SiHa cells. The protein levels of cleaved PARP and caspase 3, both apoptosis indicator proteins [6, 24], were increased with increasing MOI (parasite burden) at 6 h after infection; they were also apparently increased from 2 h after infection in *T. vaginalis*-infected SiHa cells and increased further in a infection time-dependent manner (Fig. 2a). In addition to western blotting, flow cytometric analysis confirmed that both early and late apoptosis were significantly increased in *T. vaginalis-*infected cells in an infection time-dependent manner (Fig. 2b). Nucleus fragmentation and cellular shrinkage, a characteristic morphological change of apoptosis [6], were found in *T. vaginalis*-infected cells at 2 and 6 h after infection by DAPI staining; however, uninfected cells (0 h) grew well with a distinctly complete nucleus (Fig. 1b, d). These results suggested that *T. vaginalis* induces apoptosis in SiHa cells in a MOI- and infection time-dependent manner.

Based on the results shown in Fig. 1d, e, we evaluated whether ROS are involved in the induction of apoptosis in *T. vaginalis-*infected SiHa cells. We therefore pretreated cells with the ROS scavenger NAC and then evaluated the expression of apoptosis-related proteins and MMP. We first determined the cytotoxic effects of NAC on cell viability using the MTS assay. Treatment of the SiHa cells with 0.2–1 mM NAC for 6 h elicited no significant differences in cell viability compared with cells in the medium only-treated group; however, cells treated with 5 mM NAC for 6 h showed slightly reduced viability (see Additional file 2: Figure S2a). Both cellular and mitochondrial ROS production were significantly suppressed in the *T*. *vaginalis*-infected cells following NAC pretreatment in a concentration-dependent manner (see Additional file 3: Figure S3a, b).

As shown in Fig. 2c, in the NAC-pretreated SiHa cells, *T*. *vaginalis*-induced cleavages of PARP and caspase-3 were significantly suppressed in a dose-dependent manner. The permeabilization of the mitochondrial outer membrane is a crucial step in the progression of mitochondrial apoptosis [6, 7]; thus, we also investigated whether the *T. vaginalis*-induced ROS-dependent apoptosis in SiHa cells was related to the mitochondrial apoptotic pathways. After *T. vaginalis* infection, JC-1 staining, an indicator of MMP [25], revealed a notable decrease in MMP levels, as indicated by the reduction in red fluorescence signal and an increase in green JC-1 fluorescence signal, in a parasite burden- and infection time-dependent manner compared with the signals in untreated control cells (Fig. 2d). However, these changes in JC-1 dye fluorescence were suppressed by NAC pretreatment in a dose-dependent manner (Fig. 2e). Collectively, these results suggested that *T. vaginalis* induces mitochondrial apoptosis through ROS and that ROS act upstream of PARP and caspase-3 during apoptosis induction.

### *Trichomonas vaginalis*-induced ER stress response and ER stress-mediated apoptosis in SiHa cells

**Fig. 3:**
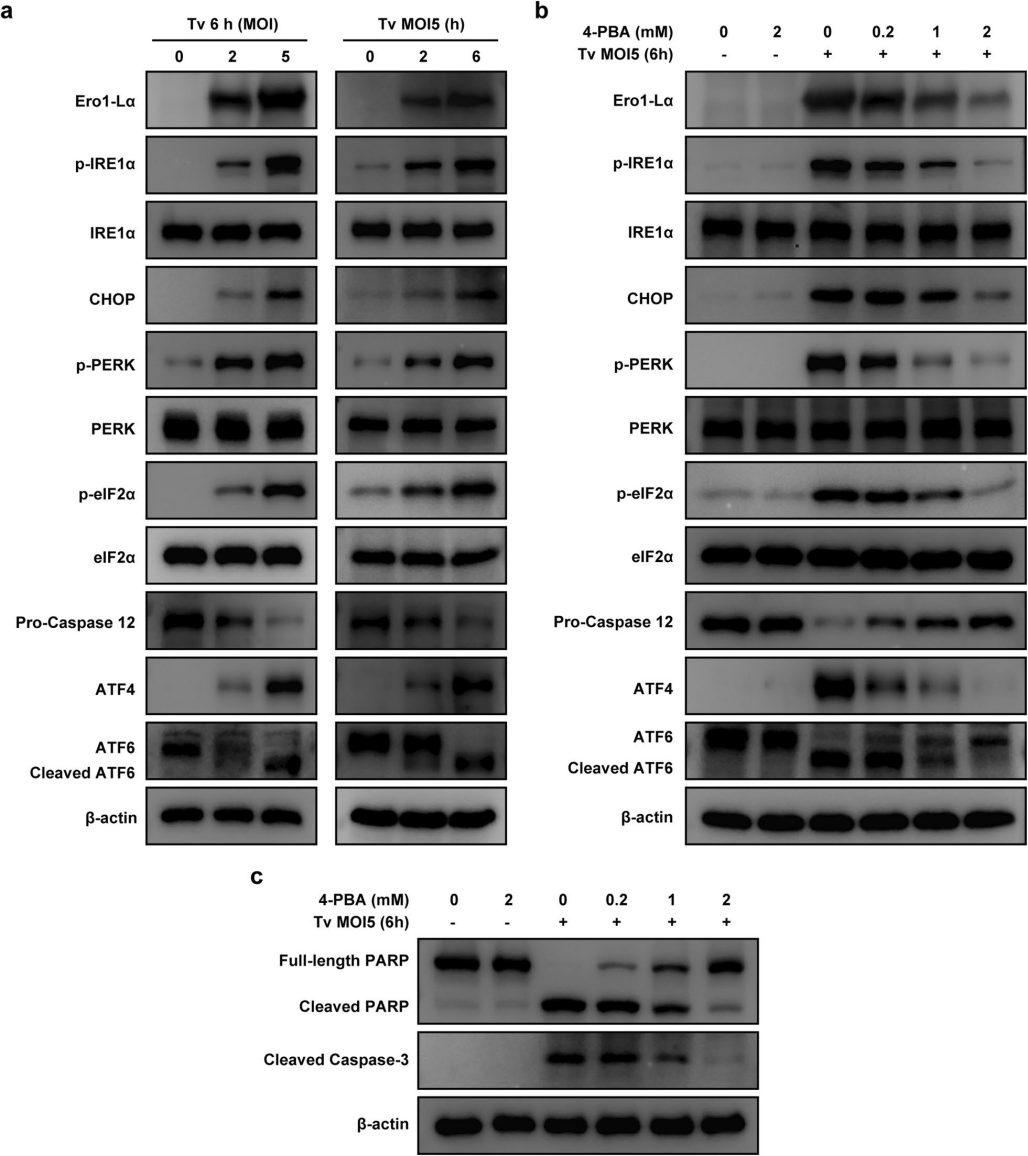
*Trichomonas vaginalis*-induced ER stress response and ER stress-dependent apoptosis in SiHa cells. **a** SiHa cells were infected with *T. vaginalis* under the indicated conditions, and ER stress-related proteins were evaluated by western blotting analysis. **b**, **c** SiHa cells were pretreated with various concentrations of an ER stress inhibitor (4-PBA) for 2 h and subsequently infected with *T. vaginalis* at MOI 5 for 6 h. The ER stress-related (**b**) and apoptosis-related (**c**) protein levels were assessed using western blotting analysis. Anti-β-actin was used as an internal control. The data shown are representative of three independent experiments with similar results

Endoplasmic reticulum stress is known to contribute to apoptosis [19, 20]. To check whether *T. vaginalis* causes ER stress in SiHa cells, we first measured the activities of three major ER stress sensor proteins in *T. vaginalis*-infected SiHa cells. As shown in Fig. 3a and Additional file 1: Figure S1b, the expression levels of phosphorylated IRE1α and PERK as well as of cleaved ATF6 were significantly increased in a parasite burden- and infection time-dependent manner. The downstream cascades of PERK, including eIF2α, ATF4 and CHOP, were also activated by *T. vaginalis* infection. Caspase-12 was consistently activated, whereas the protein level of procaspase-12 was decreased. In addition, Ero1-Lα, a luminal glycoprotein that plays a role in the formation of disulfide bonds of secreted proteins and membrane proteins [18], was also upregulated in response to *T. vaginalis* infection.

Next, to confirm whether *T*. *vaginalis-*induced apoptosis is mediated by ER stress in SiHa cells, the cells were pretreated with the ER stress inhibitor 4-phenylbutyric acid (4-PBA). 4-PBA is a low-molecular-weight fatty acid that prevents misfolded protein aggregation by interacting with hydrophobic regions in unfolded proteins [26]. There was no difference in cell viability between the SiHa cells treated with 2 mM 4-PBA for 6 h and cell in the untreated control group (see Additional file 2: Figure S2b). The expressions of ER stress-related proteins were reversed in the 4-PBA-pretreated *T*. *vaginalis*-infected SiHa cells in a dose-dependent manner as compared with those in the untreated *T*. *vaginalis*-infected cells (Fig. 3b). Surprisingly, 4-PBA pretreatment attenuated the levels of cleaved PARP and caspase-3 in *T*. *vaginalis*-infected SiHa cells in a dose-dependent manner (Fig. 3c). These results demonstrated that *T*. *vaginalis* induces ER stress response as well as ER stress-mediated apoptosis in SiHa cells.

### *Trichomonas vaginalis*-induced ROS-dependent ER stress responses in SiHa cells

**Fig. 4:**
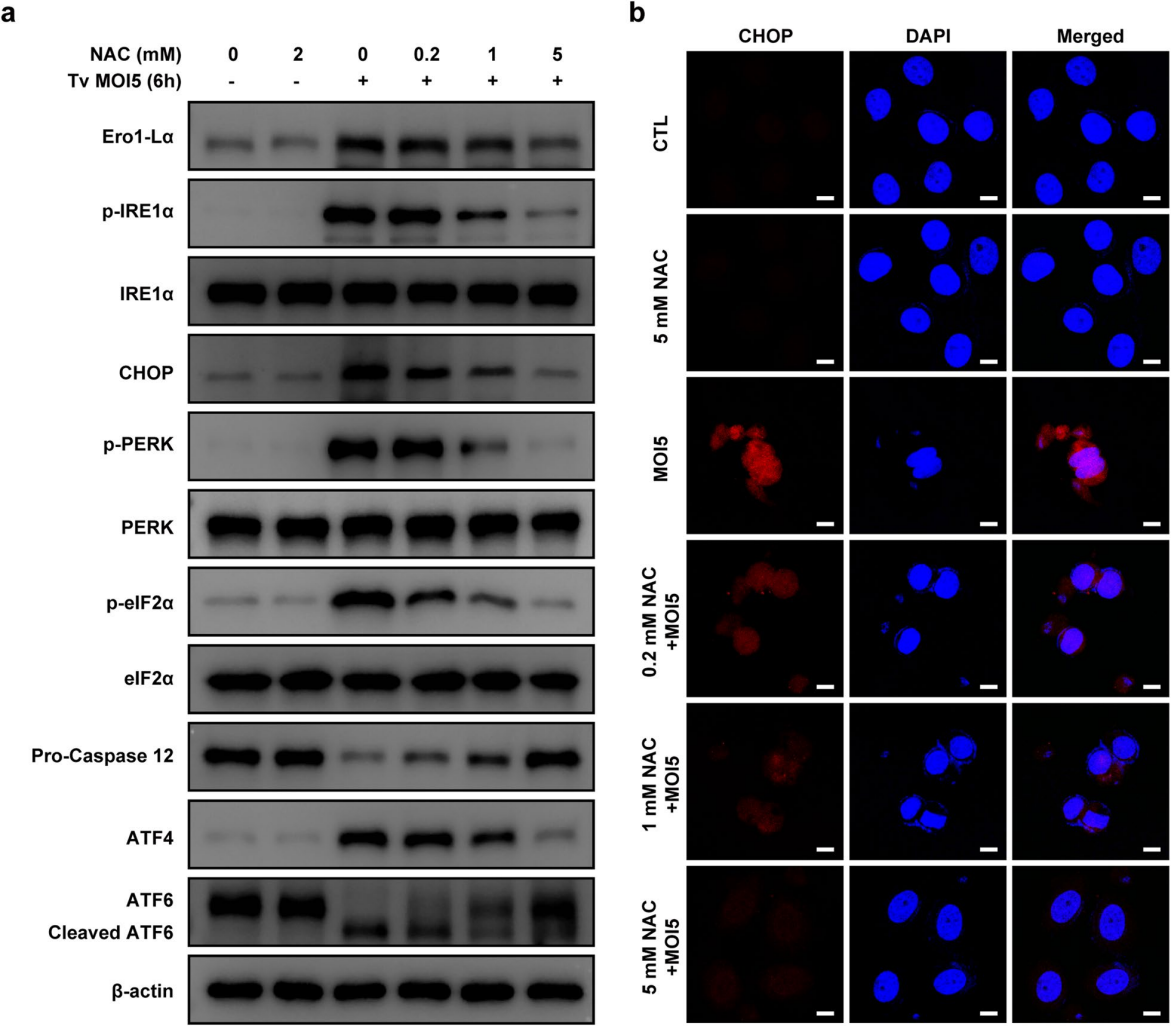
*Trichomonas vaginalis*-induced ROS-dependent ER stress response in SiHa cells. SiHa cells were pretreated with various concentrations of NAC for 2 h and subsequently infected with *T. vaginalis* at MOI 5 for 6 h. **a** The ER stress-related protein levels were assessed using western blotting analysis. Anti-β-actin was used as a loading control. **b** Representative images obtained by confocal fluorescence microscopy stained with CHOP antibody. The data shown are representative of three independent experiments with similar results. Scale bars: 10 µm

Endoplasmic reticula and mitochondria are closely related, both structurally and functionally [20]. Thus, to evaluate the effects of mitochondrial ROS in the induction of ER stress response in *T*. *vaginalis*-infected SiHa cells, we pretreated the SiHa cells with NAC and investigated the expression of ER stress-related proteins. Pretreatment with NAC significantly reduced the expression of Ero1-Lα, p-IRE1α, CHOP, p-PERK, p-elF2α, ATF4, and cleaved ATF6 in the *T*. *vaginalis*-infected SiHa cells in a dose-dependent manner, whereas the expression level of procaspase-12 was increased (Fig. 4a). Confocal microscopy imaging also confirmed that NAC pretreatment reduced *T*. *vaginalis*-induced upregulation of CHOP [also known as DNA damage-inducible gene 153 (GADD153)] in a dose-dependent manner (Fig. 4b). These results indicated that *T*. *vaginalis* induces ER stress response through mitochondrial ROS in SiHa cells, which leads to apoptosis.

### *Trichomonas vaginalis*-induced ER stress-mediated mitochondrial dysfunction in SiHa cells

**Fig. 5:**
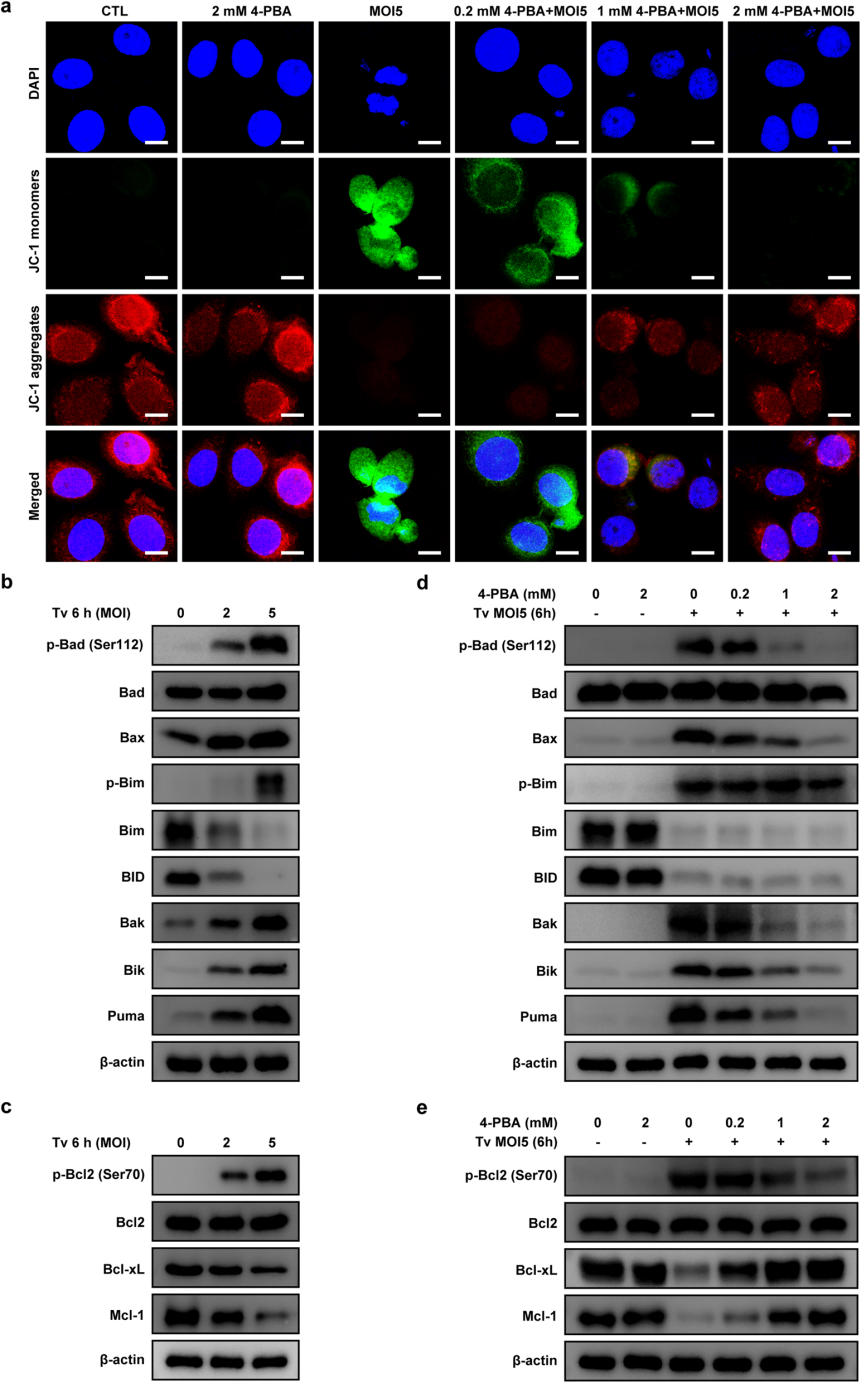
*Trichomonas vaginalis*-induced ER stress-dependent mitochondrial dysfunction in SiHa cells. **a** SiHa cells were pretreated with various concentrations of 4-PBA for 2 h and subsequently infected with *T. vaginalis* at MOI 5 for 6 h. SiHa cells were stained with JC-1, and fluorescence was detected under a confocal microscope. The figure shows representative confocal images of JC-1 aggregate (red) and monomer (green) fluorescence, respectively. **b**, **c** SiHa cells were infected with *T. vaginalis* at MOI 2 and 5 for 6 h. Pro-apoptosis Bcl-2 (**b**) and pro-survival Bcl-2 (**c**) family members were evaluated by western blotting. **d**, **e** SiHa cells were pretreated with various concentrations of 4-PBA for 2 h and subsequently infected with *T. vaginalis* at MOI 5 for 6 h. Pro-apoptosis Bcl2 (**d**) and pro-survival Bcl-2 (**e**) family members were evaluated by western blotting. Anti-β-actin was used as a loading control. The data shown are representative of three independent experiments with similar results. Scale bars: 10 µm

We also investigated whether ER stress affects mitochondrial functions in *T. vaginalis*-infected SiHa cells. Members of the Bcl-2 family proteins are major regulators of mitochondrial integrity and mitochondria-dependent caspase activation, and MMP is the key indicator of mitochondrial apoptosis [6, 7, 25]. Therefore, we investigated mitochondrial functions, including alternation of MMP and Bcl-2 family members, in *T. vaginalis*-infected SiHa cells. JC-1 dye staining revealed that *T. vaginalis* induced MMP depolarization in the SiHa cells; however, these changes in fluorescence were suppressed by 4-PBA pretreatment (Fig. 5a).

The expressions of p-Bad (Ser112), Bax, Bak, Bik and Puma were significantly increased in the *T. vaginalis*-infected SiHa cells (Fig. 5b), whereas the Bcl-xL and Mcl-1 levels were decreased in a parasite burden-dependent manner (Fig. 5c). The expression levels of p-Bad (Ser 112), Bax, Bak Bik, and Puma were significantly decreased in the 4-PBA-pretreated *T. vaginalis*-infected SiHa cells in a dose-dependent manner (Fig. 5d), whereas the levels of Bcl-xL and Mcl-1 proteins were increased (Fig. 5e).

These data indicated that MMP depolarization and Bcl-2 family protein imbalance were recovered in* T. vaginalis*-infected SiHa cells after treatment with 4-PBA. Thus, our results clearly indicated that *T. vaginalis* induces mitochondrial dysfunction via ER stress in SiHa cells, leading to apoptosis.

### *Trichomonas vaginalis*-induced ER stress-mediated mitochondrial apoptosis via the IRE1/ASK1/JNK/Bcl-2 family protein pathways in SiHa cells, dose-dependently

**Fig. 6:**
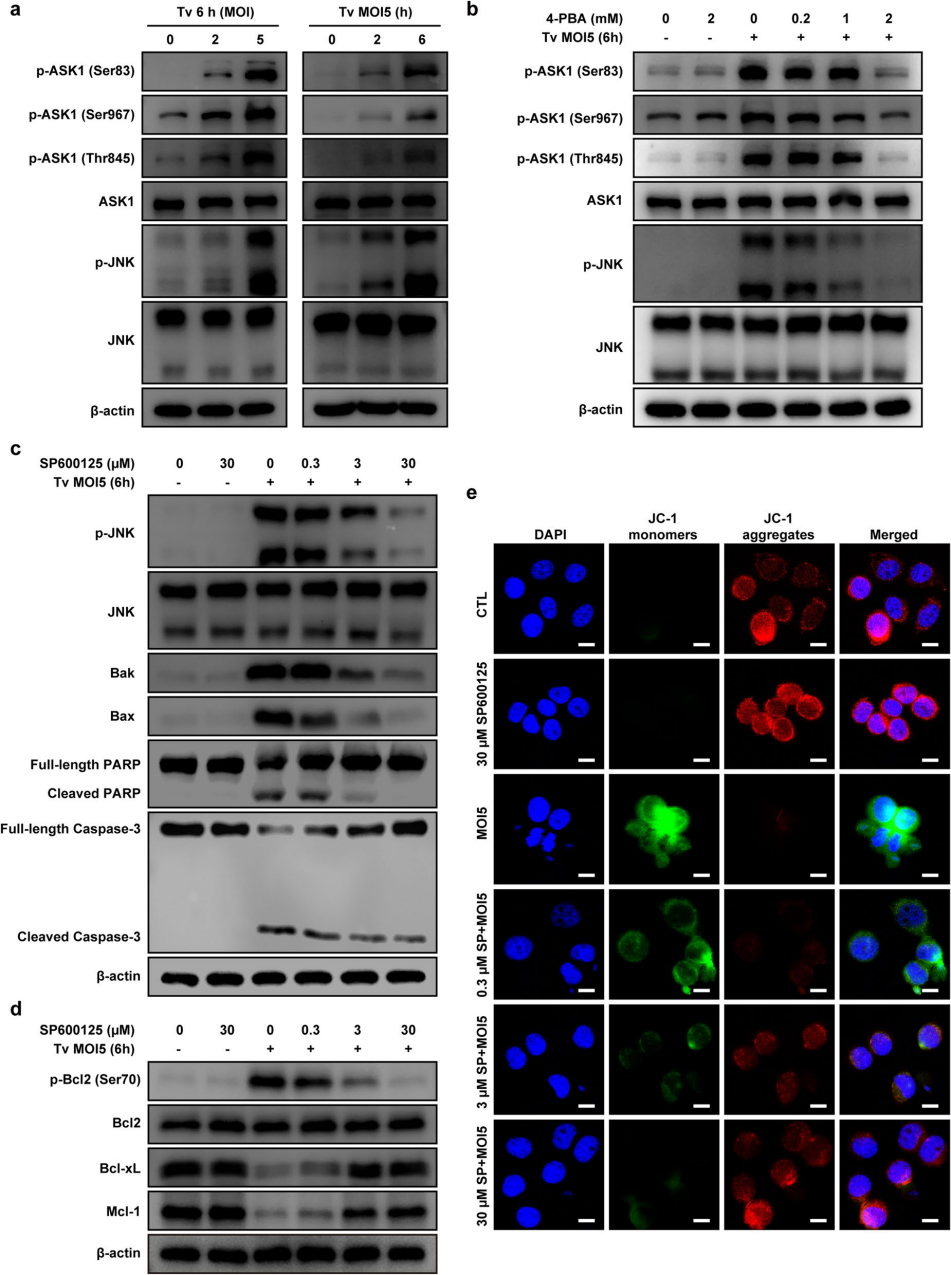
*Trichomonas vaginalis*-induced ES stress-dependent mitochondrial apoptosis via ASK1/JNK pathways in SiHa cells. **a** SiHa cells were infected with *T. vaginalis* at the indicated MOI for 6 h or at MOI 5 for the indicated time. Cells were lysed, and the ASK1/JNK phosphorylation levels were assessed by western blot analysis. **b** SiHa cells were pretreated with various concentrations of 4-PBA for 2 h and subsequently infected with *T. vaginalis* at MOI 5 for 6 h. ASK1/JNK phosphorylation levels were assessed by western blot analysis. **c**–**e** SiHa cells were pretreated with various concentrations of a JNK inhibitor (SP600125) for 2 h and subsequently infected with *T. vaginalis* at MOI 5 for 6 h. The protein levels of p-JNK, JNK, Bak, Bax, PARP and cleaved caspase 3 (**c**) and p-Bcl-2 (Ser70), Bcl-2, Bcl-xL and Mcl-1 (**d**) were assessed using western blotting analysis. Anti-β-actin was used as a loading control. JC-1 staining was observed by confocal imaging (**e**). All the results presented are representative of three independent experiments with similar results. Scale bars: 10 µm

Previous studies have reported that the IRE1/ASK1/JNK signaling cascade is one of the main downstream targets for the regulation of ER stress-induced cell apoptosis [19, 20]. Therefore, we investigated whether *T. vaginalis* activates ER stress-dependent mitochondrial apoptosis through the ASK1/JNK pathway in SiHa cells. We found a substantial increase in the phosphorylation levels of both ASK1 (Ser83, Ser967 and Thr845) and JNK in the *T. vaginalis*-infected SiHa cells in a parasite burden- and infection time-dependent manner (Fig. 6a). First, we investigated whether ASK1/JNK activation was related to ER stress in the *T. vaginalis*-infected SiHa cells. Western blotting analysis revealed that 4-PBA pretreatment attenuated the *T. vaginalis*-induced elevation of ASK1 and JNK phosphorylation levels in a dose-dependent manner (Fig. 6b). These data indicated that the *T. vaginalis*-induced ER stress activates the ASK1/JNK pathway in SiHa cells.

Next, we investigated whether the ASK1/JNK/Bcl-2 family protein pathway is associated with mitochondrial apoptosis in *T. vaginalis*-infected SiHa cells. SiHa cells were preincubated with the JNK inhibitor SP600125, and then MMP, Bcl-2 family proteins and apoptosis-related proteins were investigated. The SiHa cells treated with 30 μM SP600125 for 6 h did not significantly differ from cells in the untreated control group in terms of cell viability (see Additional file 2: Fig. S2c). Western blotting analysis revealed that Bak, Bax, cleaved PARP and caspase-3 levels were decreased in a dose-dependent manner in the SP600125-pretreated *T. vaginalis*-infected SiHa cells (Fig. 6c). In contrast, Bcl-xL and Mcl-1 levels were increased by pretreatment with SP600125 in the *T. vaginalis*-infected SiHa cells in a dose-dependent manner (Fig. 6d). In addition, SP600125 pretreatment substantially increased MMP levels in the *T. vaginalis*-infected SiHa cells, as demonstrated by increased red and reduced green JC-1 fluorescence, in a dose-dependent manner (Fig. 6e). These observations provided evidence that *T. vaginalis* induces ER stress-mediated mitochondrial apoptosis via the IRE1/ASK1/JNK/Bcl-2 family protein signaling pathways in SiHa cells.

### *Trichomonas vaginalis* excretory/secretory product (ESP) induced mitochondria ROS, apoptosis and ER stress response in SiHa cells

**Fig. 7:**
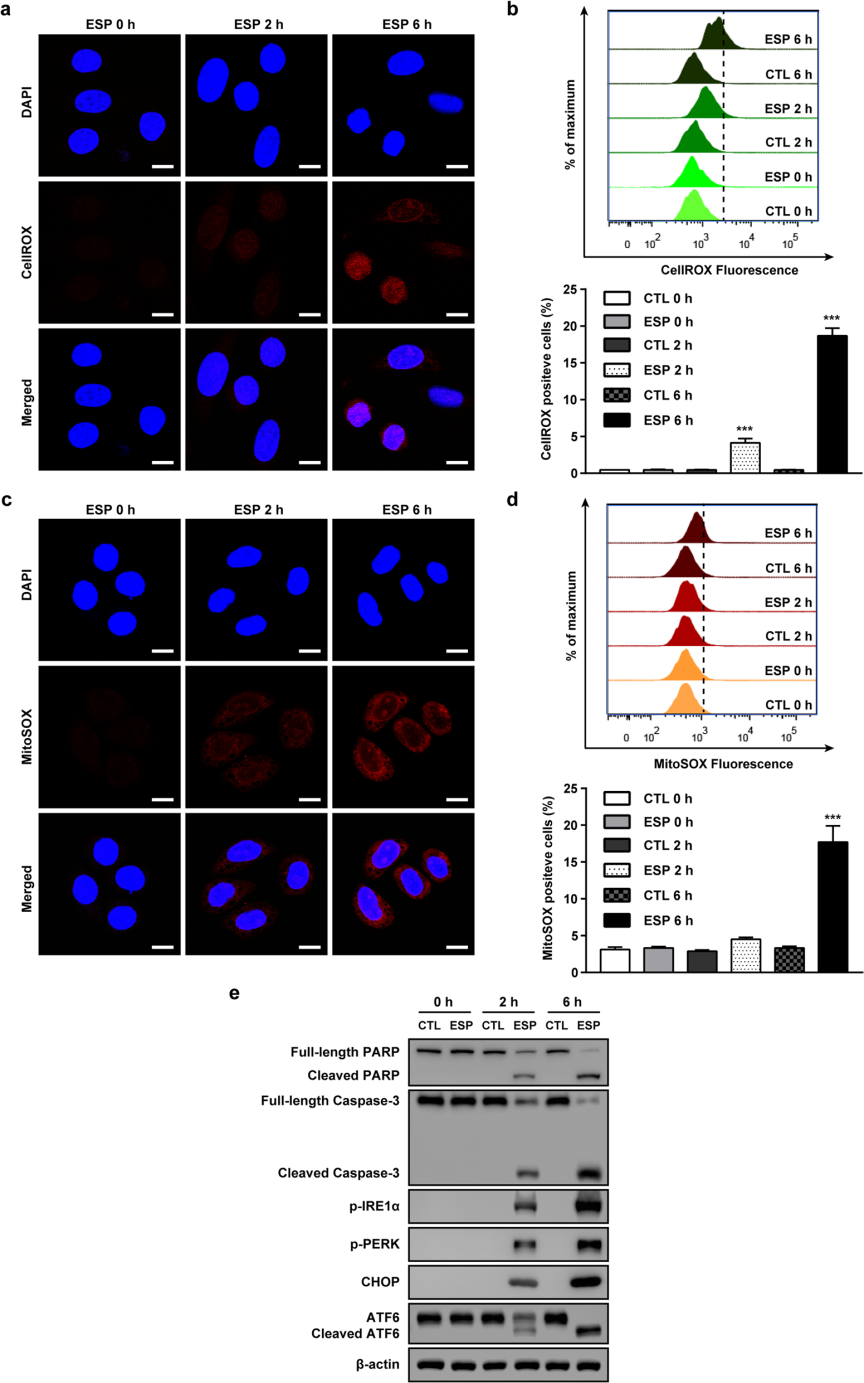
*Trichomonas vaginalis* ESP induced apoptosis and ER stress response and generated mitochondrial ROS production in SiHa cells. SiHa cells were treated with 100 μg/ml *T. vaginalis* ESP for 0, 2 or 6 h. **a**, **b** Cellular ROS production was measured by confocal microscopy (**a**) and flow cytometry (**b**) with the CellROX oxidative stress reagent, a fluorogenic probe for measuring oxidative stress in live cells. Plots depict the CellROX-positive cells as determined by fluorescence analysis of flow cytometry. Asterisks indicate significant difference (***P < 0.001) in mean fluorescence compared with the untreated control cells under the same conditions. **c**, **d** Mitochondrial ROS production was determined by confocal microscopy (**c**) and flow cytometry (**d**) with MitoSOX, a mitochondrial ROS dye. Plots depict the MitoSOX-positive cells as determined by fluorescence analysis of flow cytometry. Data shown are representative of three independent experiments with similar results. Asterisks indicate significant difference (***P < 0.001) in mean fluorescence compared with the untreated control cells under the same conditions. **e** Apoptosis- and ER stress-related protein expression levels were evaluated by western blot. The data shown are representative of three independent experiments with similar results. Scale bars: 10 µm. Abbreviations: CTL, Untreated control SiHa cells; ESP, SiHa cells treated with *T. vaginalis* ESP

*Trichomonas vaginalis* is an extracellular protozoan parasite; it adheres to host cells and hydrolyzes organic components of host cells for survival, which is its mode of infection and pathogenesis [27–29]. There have been several reports of the ESP of *T. vaginalis* having cytotoxic effects on host cells [12, 14, 30]. Thus, we evaluated whether *T. vaginalis* ESP can induce the mitochondrial ROS production, ER stress response and apoptosis in SiHa cells. Cellular ROS levels were significantly increased in SiHa cells by *T. vaginalis* ESP from 2 h after treatment and increased further at 6 h after treatment (Fig. 7a, b). Similarly, mitochondrial ROS levels were also significantly increased after treatment with *T. vaginalis* ESP in an incubation time dependently (Fig. 7c, d).

Next, we evaluated the expressions of apoptosis- and ER stress-related proteins in SiHa cells after treatment with *T. vaginalis* ESP. The protein levels of cleaved PARP and caspase 3 significantly increased in cells from 2 h after *T. vaginalis* ESP treatment, and further increased at 6 h post-treatment (Fig. 7e). Similarly, the protein levels of p-IRE1α, CHOP, p-PERK and cleaved ATF6 were significantly increased in cells after treatment with *T. vaginalis* ESP, incubation in a time-dependent manner (Fig. 7e). The results suggest that ESP from live *T. vaginalis* induce mitochondrial ROS production, apoptosis and ER stress response in the SiHa cells in an incubation time dependent manner.

## Discussion

*Trichomonas vaginalis* primarily causes lesions in the vagina and cervix in women [1]. SiHa is a human cervical cancer cell line that is used as an in vitro model for cervical cancer and cervicovaginal infection, such as papilloma virus [31], *Candida* [32] and *Trichomonas* infection [9, 12]. Thus, we used the SiHa cell line as a host cell to investigate the pathogenesis of human trichomoniasis. In this study, we investigated the involvement of ER stress in apoptosis induction in *T*. *vaginalis*-infected SiHa cells and its molecular mechanisms. *Trichomonas vaginalis* induced mitochondrial apoptosis through ROS in SiHa cells and also induced ER stress response directly and in an ROS-dependent manner in SiHa cells. In addition, *Trichomonas vaginalis* induced ER stress-mediated apoptosis in SiHa cells through the PERK/CHOP and IRE1/ASK1/JNK/Bcl-2 family protein pathways. This study is the first to demonstrate ER stress involvement in apoptosis induction in *T. vaginalis*-infected mammalian cells via the ROS–ER stress–mitochondrial apoptosis pathways.

We and other researchers have previously reported that *T. vaginalis* induces apoptosis in various cells [8–14] and that ER stress is involved in apoptosis induction via various agents, including some pathogens [21–23]. In this study, we also confirmed the induction of mitochondrial apoptosis in *T. vaginalis*-infected SiHa cells by western blotting and flow cytometry. We then checked whether *T. vaginalis* infection induces ER stress responses in SiHa cells. Perturbation of the physiological status of the ER triggers an ER-related stress response or UPR, which is distinguished by the action of three signaling proteins: IRE1α, PERK and ATF6 [18, 19]. We found that *T*. *vaginalis* infection increased the protein levels of p-IRE1α, p-PERK, ATF4, cleaved ATF6 and CHOP and decreased the levels of procaspase-4 and procaspase-12 in SiHa cells. These results demonstrate that *T. vaginalis* has the capacity to induce an ER stress response in SiHa cells. However, pretreatment with the ER stress inhibitor 4-PBA was found to significantly attenuate the levels of ER stress-related proteins in *T*. *vaginalis*-infected cells. Furthermore, 4-PBA pretreatment attenuated the cleavages of PARP and caspase-3 proteins in *T*. *vaginalis*-infected SiHa cells in a dose dependent manner. These findings indicate that ER stress and apoptosis interact with each other and that ER stress plays a pivotal role in apoptosis induction in *T*. *vaginalis*-infected SiHa cells, similar to that observed in *Toxoplasma gondii*-infected neural cells [33] and *Leishmania infantum*-infected macrophages [34]. ER stress-mediated apoptosis may be caused by activation of the ER-specific cysteine protease caspase-12 or transcription factor CHOP [18, 19].

Having established the involvement of ER stress in apoptosis induction in *T. vaginalis*-infected SiHa cells, we subsequently examined the role of ROS in promoting ER stress. Several studies have shown that ROS generation is one of the most critical factors for apoptosis induction in various mammalian cells infected with pathogens [8, 9, 21]. ROS-dependent caspase-3 activation plays an important role in the induction of apoptosis induction of human neutrophils induced by *T. vaginalis* [8]. It has been shown that the ER communicates with mitochondria via calcium and ROS signaling [19, 20]. In the present study, *T*. *vaginalis* induced mitochondrial ROS and ER stress responses in SiHa cells, and pretreatment with NAC or 4-PBA significantly suppressed mitochondrial ROS production, along with the expression levels of apoptosis- and ER stress-related proteins, in *T*. *vaginalis*-infected cells. These observations provide evidence of apoptotic crosstalk signaling between the ER and mitochondria via ROS, thereby indicating that *T*. *vaginalis* induces both ROS-dependent mitochondrial apoptosis and ROS-dependent ER stress-mediated apoptosis in SiHa cells. Similar findings were also reported in previous studies, in which *Mycobacterium avium* was shown to induce apoptosis via an ROS-dependent ER stress response in macrophages [21], and silibinin, a biologically active compound of milk thistle, was found to induce ER stress-mediated mitochondrial ROS-dependent apoptosis in prostate cell lines [35].

ER and mitochondria have been identified as key players in the sensing and perception of cellular stress [20]. Based on the aforementioned observations, we proceeded to investigate whether ER stress mediates mitochondrial apoptotic pathways in *T. vaginalis*-infected SiHa cells. Mitochondria and the ER play important roles in the regulation and transmission of apoptotic signals, with the regulatory mechanisms determined by a balance among Bcl-2-family proteins [20, 36]. Under severe and sustained ER stress conditions, the intrinsic apoptotic pathway can be activated by the IRE1-mediated activation of tumor necrosis factor receptor-associated factor 2 (TRAF2), which in turn stimulates the ASK1/JNK kinase cascade [19, 37]. In the present study, we found that *T*. *vaginalis* induced ASK1 and JNK phosphorylation in SiHa cells, whereas pretreatment with either 4-PBA or SP600125 contributed to a reduction in the levels of p-IRE1α, cleaved caspase-3, cleaved PARP, p-ASK1 and p-JNK proteins in *T. vaginalis*-infected SiHa cells. In addition, pretreatment with 4-PBA or SP600125 was found to enhance MMP, Bcl-xl, and Mcl-1 protein levels but reduce Bax and Bak protein levels in the infected cells. These results indicate that *T*. *vaginalis* induces apoptosis through ER stress-mediated mitochondrial dysfunction in SiHa cells via the IRE1/ASK1/JNK/Bcl-2 family protein pathways, and finally led to apoptosis. These findings can be explained in part by the fact that Bax and Bak are localized to both the mitochondria and ER and that their overexpression promotes apoptotic calcium mobilization during ER-mediated mitochondrial cell death [38]. A similar apoptotic mechanism has been proposed for endothelial cells treated with silica nanoparticles, in which apoptosis was induced via ER stress-associated IRE1/JNK signaling to the mitochondria mediated by Bcl-2 family members [39].

We also considered the cytopathogenic factors in *T. vaginalis*-infected cells. *Trichomonas vaginalis* is an extracellular protozoan parasite that produces a variety of proteases, such as cysteine proteases and metalloproteinases, which are important regulators of pathogenesis, invasion and survival [10–12, 27–29]. In previous studies, we and other researchers have reported that the ESP of live *T. vaginalis*, which comprises proteases, play critical roles in the induction of apoptosis in *T. vaginalis*-infected cells [9, 12, 14]. In the present study, we confirmed the induction of mitochondrial ROS production, apoptosis and ER stress responses in SiHa cells after treatment with *T. vaginalis* ESP, the results of which were consistent with those observed in live *T. vaginalis*-infected cells. We assume that these effects can be attributed to the interaction between *T. vaginalis* ESP and the surface proteins of host cells, which subsequently induce a range of pathogenetic events, such as ROS production, ER stress response and cell death [12, 14, 30]. However, the findings of a few studies have indicated that in human trichomoniasis, the death of host cells can also proceed via necrosis rather than the induction of apoptosis [28], and the difference between these results could be attributed to differences in the cell lines used, parasite strains, adherence ability and/or culture conditions.

## Conclusions

**Fig. 8:**
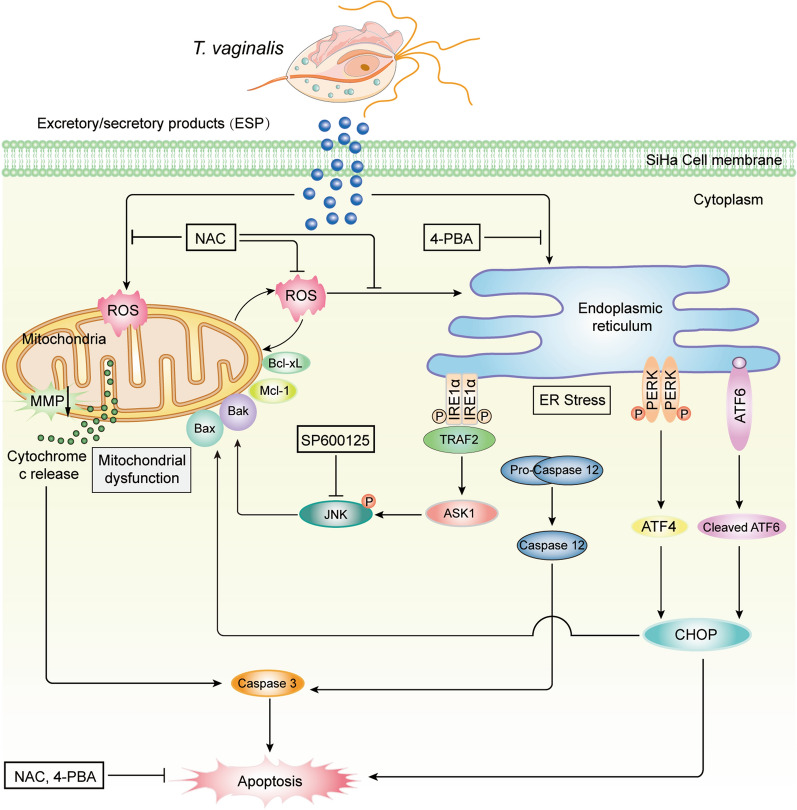
Schematic model of apoptosis by *T. vaginalis* in SiHa cells. *Trichomonas vaginalis* induces apoptosis through mitochondrial ROS and ER stress response, and also induces ROS-dependent ER stress response and ER stress-mediated mitochondrial dysfunction through the IRE1/ASK1/JNK/Bcl-2 family protein pathways, finally leading to apoptosis of the SiHa cells. In addition, *T. vaginalis* ESP also exerted mitochondrial ROS production, apoptosis and ER stress response in SiHa cells. Collectively, *T. vaginalis* infection induces apoptosis via ROS–ER stress-mitochondrial apoptosis pathways by interaction with mitochondria and ER in human cervical cancer SiHa cells, and this pathogenesis may be involved in the ESP released from live *T. vaginalis*

Our results showed that live *T. vaginalis* induced apoptosis through mitochondrial ROS and ER stress responses and promoted the ER stress response both directly and in an ROS-dependent manner in human cervical cancer SiHa cells, dependent on the parasite burden and infection time. In addition, *T. vaginalis* induced ER stress-mediated mitochondrial dysfunction and finally led to apoptosis of SiHa cells through the IRE1/ASK1/JNK/Bcl-2 family protein pathways. *Trichomonas vaginalis* ESP also promoted mitochondrial ROS production, apoptosis and the ER stress response in SiHa cells in an incubation time-dependent manner. Collectively, our results suggest that *T. vaginalis* infection induces apoptosis as well as the ER stress response via ROS–ER stress-mitochondrial apoptosis pathways by interacting with the mitochondria and ER in human cervical cancer SiHa cells, and that ER stress is one of the major factors contributing to the apoptosis of *T. vaginalis*-infected cells. These pathogenesis mechanisms may be associated with the ESP released from live *T. vaginalis* (Fig. 8). Our findings will potentially contribute to enhancing our current understanding of the molecular mechanisms underlying the pathogenesis of human trichomoniasis.

## Supplementary Information

**Additional file 1: Figure S1**. *Trichomonas vaginalis*-induced cell cytotoxicity and ER stress in SiHa cells. SiHa cells were infected with *T. vaginalis* at various MOIs (1, 2, 5 and 10) for the indicated times (0, 0.5, 2, 6, 12 and 24).** a** The percentages of LDH-dependent cytotoxicity in the medium was measured by LDH assay. The data represent the mean value ± standard deviation (SD) of at least three independent experiments. Asterisks indicate significant difference (**P < 0.01, ***P < 0.001) compared with untreated control cells under the same conditions.** b** The levels of apoptosis- and ER stress-related protein were measured by western blot, and anti-β-actin was used as a loading control. **c** Equal volume without quantification at each lane after collecting the* T. vaginalis*-infected SiHa cells. The expected protein loss which might be caused by apoptosis was not observed until 24 h after infection. The β-actin levels in the whole cell lysate from samples loaded with equal volume did not show any significant difference from samples loaded with equal protein amount (Fig. S1c vs. Fig. S1b)

**Additional file 2: Figure S2**. Effects of various concentrations of NAC (ROS scavenger), 4-PBA (ER stress inhibitor) and SP600125 (JNK1/2 inhibitor) on the viability of human cervical cancer SiHa cells. SiHa cells treated with 0.2, 1 and 5 mM of NAC (**a**), 0.2, 1 and 2 mM of 4-PBA (**b**) and 0.3, 3 and 30 µM of SP600125 (**c**), in 5% CO2 at 37°C for 0, 2, 6, 12 and 24 h. Cell viability was checked by the MTS assay. The data represent the mean ± SD of at least three independent experiments

**Additional file 3: Figure S3**.* Trichomonas vaginalis*-induced ROS production was suppressed with NAC pretreatment in SiHa cells. SiHa cells were pretreated with different concentrations of NAC and then infected with* T. vaginalis* at MOI 5 for 6 h.** a** Cellular ROS production was detected by CellROX. Percentages of CellROX-positive cells, as determined by densitometric analysis of fluorescence images.** b** Mitochondrial ROS production was detected by MitoSOX reagent. Percentages of MitoSOX positive cells, as determined by densitometric analysis of fluorescence images. Asterisks indicate significant differences (*P < 0.05, **P < 0.01, ***P < 0.001) compared with the untreated control cells. The data shown are representative of three independent experiments with similar results. Scale bars: 10 µm.

## Data Availability

All datasets generated during the present study are included in the published article including the Additional files.

## Abbreviations

ASK1: Apoptosis signal regulating kinase 1
ATF: Activating transcription factor
CHOP: CCAAT/enhancer-binding protein–homologous protein
DAPI: 4′,6-Diamidino-2-phenylindole
ESP: Excretory/secretory products
ER: Endoplasmic reticulum
IRE1: Inositol-requiring enzyme 1
JC-1: 5,5′,6,6′-Tetrachloro-1,1′,3,3′-tetraethyl-imidacarbocyanine iodide
JNK: C-Jun N-terminal kinases
LDH: Lactate dehydrogenase
MMP: Mitochondrial membrane potential
MOI: Multiplicities of infection
MTS: 3-(4,5-Dimethylthiazol-2-yl)-5-(3-carboxymethoxyphenyl)-2-(4-sulfophenyl)-2H-tetrazolium, inner salt
NAC: *N*-Acetyl cysteine
PARP: Poly (ADP‐ribose) polymerase
4-PBA: 4-Phenylbutyric acid
PERK: Protein kinase RNA-like ER kinase
ROS: Reactive oxygen species
TRAF2: Tumor necrosis factor receptor-associated factor 2
UPR: Unfolded protein response
