# Supplementary material for: Trichomonas vaginalis induces apoptosis via ROS and ER stress response through ER–mitochondria crosstalk in SiHa cells
Source: Parasit Vectors. 2021 Dec 11;14:603. doi: 10.1186/s13071-021-05098-2 (PMC8665556; doi:10.1186/s13071-021-05098-2)
Supplement: Supplementary file 1 — Additional file 1: Figure S1. Trichomonas vaginalis-induced cell cytotoxicity and ER stress in SiHa cells. SiHa cells were infected with T. vaginalis at various MOIs (1, 2, 5 and 10) for the indicated times (0, 0.5, 2, 6, 12 and 24). a The percentages of LDH-dependent cytotoxicity in the medium was measured by LDH assay. The data represent the mean value ± standard deviation (SD) of at least three independent experiments. Asterisks indicate significant difference (**P < 0.01, ***P < 0.001) compared with untreated control cells under the same conditions. b The levels of apoptosis- and ER stress-related protein were measured by western blot, and anti-β-actin was used as a loading control. c Equal volume without quantification at each lane after collecting the T. vaginalis-infected SiHa cells. The expected protein loss which might be caused by apoptosis was not observed until 24 h after infection. The β-actin levels in the whole cell lysate from samples loaded with equal volume did not show any significant difference from samples loaded with equal protein amount (Fig. S1c vs. Fig. S1b) [file 13071_2021_5098_MOESM1_ESM.docx]

**
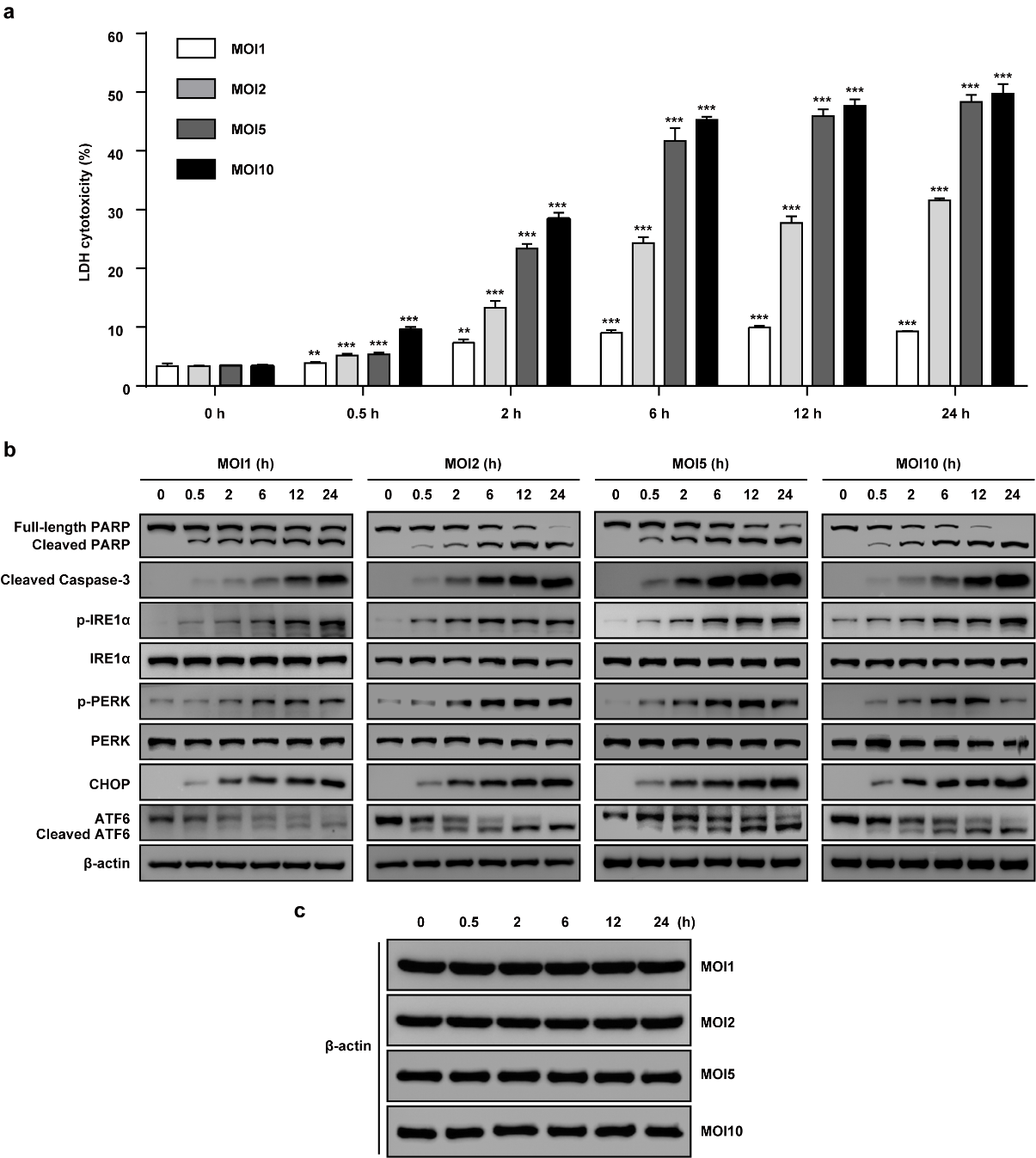
**

**Fig. S1.** Analysis of cytotoxicity and expressions of apoptosis- and ER stress-related proteins in *T. vaginalis*-infected SiHa cells at various conditions. *T. vaginalis* induced cell cytotoxicity and ER stress in SiHa cells. SiHa cells were infected with *T. vaginalis* at various MOIs (1, 2, 5 and 10) for the indicated times (0, 0.5, 2, 4, 6, 12 and 24). (**a**) The percentages of LDH-dependent cytotoxicity in the medium was measured by LDH assay. The data represent the mean value ± standard deviations (SD) of at least three independent experiments. ****P*<0.001, compared with untreated control cells under the same conditions. (**b, c**) The levels of apoptosis- and ER stress-related protein were measured by western blot, and anti-β-actin was used as a loading control. Expression of β-actin protein was observed in the samples loaded with equal protein amount (**b**) or equal volume without quantification (**c**) at each lane after collecting the *T. vaginalis*-infected SiHa cells. The expected protein loss which might be caused by apoptosis was not observed until 24 h after infection. The β-actin levels in the whole cell lysate from samples loaded with equal volume did not show any significant difference from samples loaded with equal protein amount (Fig. S1**c** versus Fig. S1**b**).
