# Supplementary material for: Trichomonas vaginalis induces apoptosis via ROS and ER stress response through ER–mitochondria crosstalk in SiHa cells
Source: Parasit Vectors. 2021 Dec 11;14:603. doi: 10.1186/s13071-021-05098-2 (PMC8665556; doi:10.1186/s13071-021-05098-2)
Supplement: Supplementary file 2 — Additional file 2: Figure S2. Effects of various concentrations of NAC (ROS scavenger), 4-PBA (ER stress inhibitor) and SP600125 (JNK1/2 inhibitor) on the viability of human cervical cancer SiHa cells. SiHa cells treated with 0.2, 1 and 5 mM of NAC (a), 0.2, 1 and 2 mM of 4-PBA (b) and 0.3, 3 and 30 µM of SP600125 (c), in 5% CO2 at 37°C for 0, 2, 6, 12 and 24 h. Cell viability was checked by the MTS assay. The data represent the mean ± SD of at least three independent experiments [file 13071_2021_5098_MOESM2_ESM.docx]

**
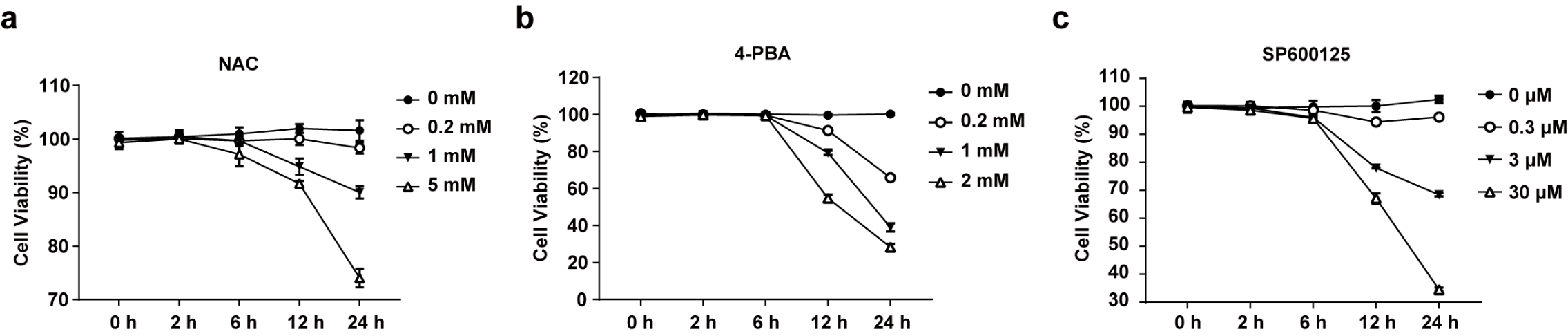
**

**Fig. S2.** Effects of various concentrations of NAC (ROS scavenger), 4-PBA (ER stress inhibitor) and SP600125 (JNK1/2 inhibitor) on the viability of human cervical cancer SiHa cells. SiHa cells treated with 0.2, 1, and 5 mM of NAC (**a**); 0.2, 1, and 2 mM of 4-PBA (**b**); and 0.3, 3, and 30 µM of SP600125 (**c**), in 5% CO_2_ at 37°C for 0, 2, 6, 12, and 24 h. Cell viability was checked by MTS assay. The data represent the mean ± SD of at least three independent experiments.
