# Supplementary material for: Trichomonas vaginalis induces apoptosis via ROS and ER stress response through ER–mitochondria crosstalk in SiHa cells
Source: Parasit Vectors. 2021 Dec 11;14:603. doi: 10.1186/s13071-021-05098-2 (PMC8665556; doi:10.1186/s13071-021-05098-2)
Supplement: Supplementary file 3 — Additional file 3: Figure S3. Trichomonas vaginalis-induced ROS production was suppressed with NAC pretreatment in SiHa cells. SiHa cells were pretreated with different concentrations of NAC and then infected with T. vaginalis at MOI 5 for 6 h. a Cellular ROS production was detected by CellROX. Percentages of CellROX-positive cells, as determined by densitometric analysis of fluorescence images. b Mitochondrial ROS production was detected by MitoSOX reagent. Percentages of MitoSOX positive cells, as determined by densitometric analysis of fluorescence images. Asterisks indicate significant differences (*P < 0.05, **P < 0.01, ***P < 0.001) compared with the untreated control cells. The data shown are representative of three independent experiments with similar results. Scale bars: 10 µm. [file 13071_2021_5098_MOESM3_ESM.docx]

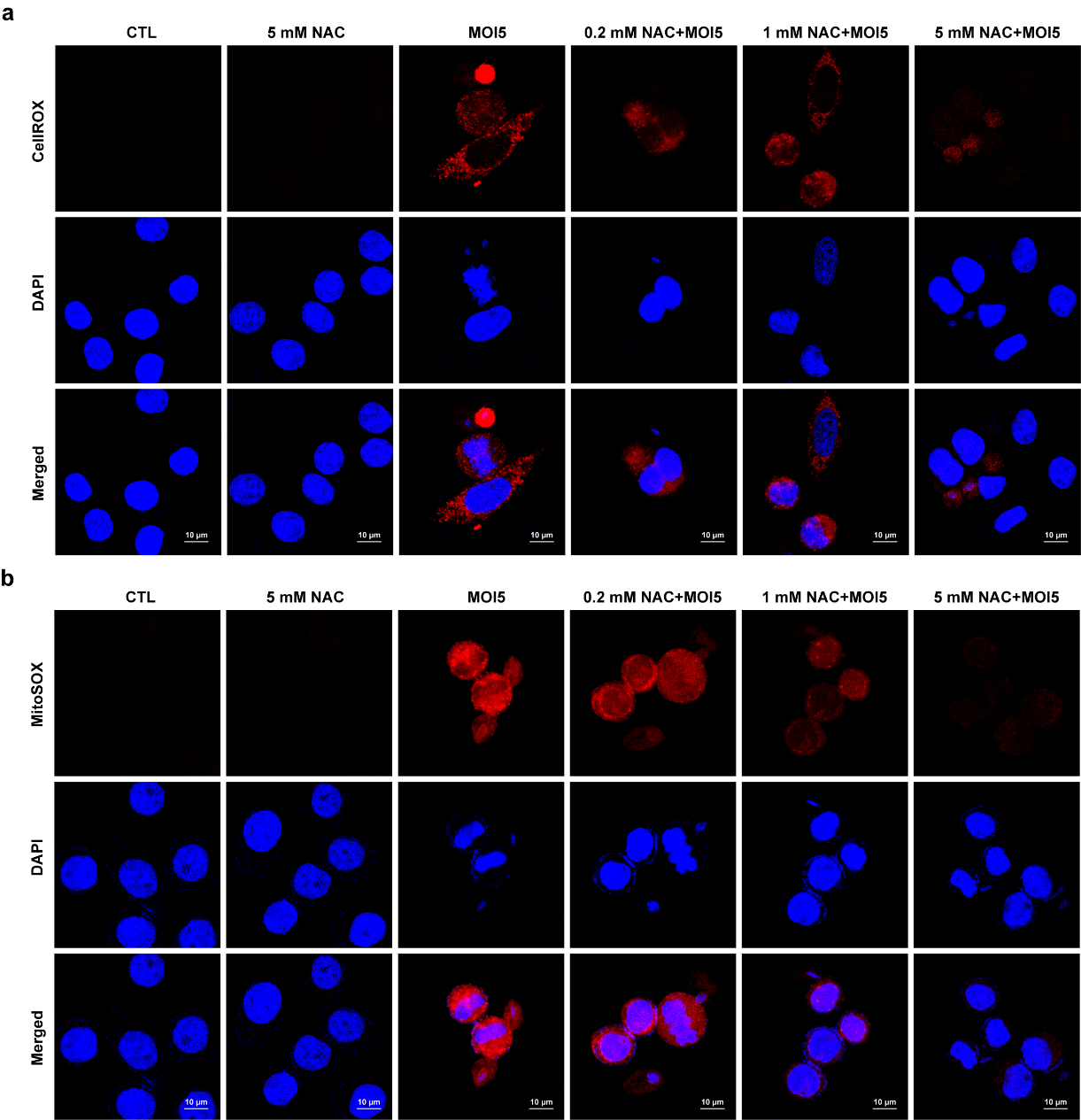


**Fig. S3.** *T. vaginlais*-induced ROS production was suppressed with NAC pretreatment in SiHa cells. SiHa cells were pretreated with numerous concentrations of NAC and then infected with *T. vaginalis* at MOI 5 for 6 h. (**a**) Cellular ROS production was detected by CellROX. Percentages of CellROX positive cells, as determined by densitometric analysis of fluorescence images. (**b**) Mitochondrial ROS production was detected by MitoSOX reagent. Percentages of MitoSOX positive cells, as determined by densitometric analysis of fluorescence images. Differences were considered significant if **P*<0.05, ***P*<0.01, or ****P*<0.001, compared with the untreated control cells. The data shown are representative of three independent experiments with similar results. Scale bars: 10 µm.
